# The plastoquinone pool of *Nannochloropsis oceanica* is not completely reduced during bright light pulses

**DOI:** 10.1371/journal.pone.0175184

**Published:** 2017-04-12

**Authors:** Gunvor Røkke, Thor Bernt Melø, Martin Frank Hohmann-Marriott

**Affiliations:** 1 Department of Biotechnology, Norwegian University of Science and Technology, Trondheim, Norway; 2 Department of Physics, Norwegian University of Science and Technology, Trondheim, Norway; University of Hyderabad School of Life Sciences, INDIA

## Abstract

The lipid-producing model alga *Nannochloropsis oceanica* has a distinct photosynthetic machinery. This organism possesses chlorophyll *a* as its only chlorophyll species, and has a high ratio of PSI to PSII. This high ratio of PSI to PSII may affect the redox state of the plastoquinone pool during exposure to light, and consequently may play a role in activating photoprotection mechanisms. We utilized pulse-amplitude modulated fluorometry to investigate the redox state of the plastoquinone pool during and after bright light pulses. Our data indicate that even very intense (5910 μmol photons s^-1^m^-2^ of blue light having a wavelength of 440 nm) light pulses of 0.8 second duration are not sufficient to completely reduce the plastoquinone pool in *Nannochloropsis*. In order to achieve extensive reduction of the plastoquinone pool by bright light pulses, anaerobic conditions or an inhibitor of the photosynthetic electron transport chain has to be utilized. The implication of this finding for the application of the widely used saturating pulse method in algae is discussed.

## Introduction

Pulse Amplitude Modulated (PAM) fluorometry has proven a valuable technique for studying the photosynthetic performance of plants and algae *in situ* [1, 2].

A PAM fluorometer assesses variable chlorophyll fluorescence by applying very weak measuring light pulses that ideally do not induce photosynthesis. The fluorescence induced by these low energy light pulses can be electronically isolated from the fluorescence induced by other light sources. Consequently, the fluorescence signal obtained by a PAM fluorometer is easily interpreted and independent of applied light sources, which could include photosynthetically active light or additional saturating light pulses. Sophisticated PAM fluorometry techniques have been developed that allow for the assessment of photosynthetic performance and for the characterization of different mechanisms that modulate chlorophyll fluorescence.

### Modulation of Chlorophyll fluorescence

The main modulator of chlorophyll fluorescence is the redox state of Q_A_, a plastoquinone that is the first stable electron acceptor of Photosystem II (PSII). From Q_A_ the electron passes to Q_B_, which is a PSII-associated member of the plastoquinone pool. If Q_A_ is oxidized, excitation energy captured in the light harvesting complexes associated with PSII (LHCII) is efficiently used for charge separation, and the fluorescence emission will thus be low [3]. However, if Q_A_ is already reduced, and therefore unable to accept an electron, excitation energy captured by chlorophylls in the light harvesting complexes will be given off as fluorescence.

A second mechanism that modulates the fluorescence yield is state transitions. If an alga or a plant receives an unusually high amount of light energy, electrons can accumulate in the electron transport chain leading to the generation of reactive oxygen species. In order to avoid the production of reactive oxygen species, some plants and algae are able to move some of their light harvesting complexes (LHCs), which are usually associated with PSII to Photosystem I (PSI). This re-balances the photosynthetic electron transport chain, as fewer electrons are generated by PSII and more electrons are being disposed of by PSI. While state transitions have been established in plants [4] and certain algae [5], state transitions are not thought to be a major contributor to modulating fluorescence in heterokont algae, such as *Nannochloropsis*.

State transitions can also be induced by oxygen depletion [6, 7]. When cells are deprived of oxygen, which is their usual mitochondrial terminal electron acceptor, they will no longer be able to re-oxidise NADH to NAD^+^ [8]. Consequently, electrons will accumulate within the cell, and eventually also reduce the PQ pool.

A third mechanism that modulates fluorescence is the conversion of certain pigments within the light harvesting complexes, enabling them to dissipate excitation as heat. Violaxanthin, which is a light harvesting xanthophyll pigment, can be converted via antheraxanthin into zeaxanthin, which is efficient in converting excess excitation energy into heat. This protective pathway is termed the xanthophyll cycle, and has been demonstrated in plants and green algae. It is also thought of as a major modulator of fluorescence in heterokont algae [9].

An additional, often neglected fluorescence modulating mechanism is the decrease in chlorophyll fluorescence yield occurring when oxidised plastoquinones interact with chlorophyll *a* molecules. This type of fluorescence modulation was termed “non-photochemical quenching of chlorophyll *a* by oxidised plastoquinone” by Haldimann and Tsimilli-Michael in 2005 [10], and was first described by Vernotte et al. in 1979 [11]. The effect of this quenching mechanism can be observed in conditions that reduce the plastoquinone pool, such as anaerobic conditions [12].

### Photochemical vs. non-photochemical quenching

A decrease in fluorescence is often termed “fluorescence quenching”, indicating that the energy captured by the light harvesting complexes is dissipated by other means than via emitting fluorescence. If excitation energy is utilized by PSII to perform a charge separation, a “photochemical quench”, mainly modulated by the availability of oxidized Q_A_, is present. A decrease in the chlorophyll fluorescence yield that is not caused by photochemistry is called non-photochemical quenching (NPQ) as a collective term. Both state-transition-dependent quenching and xanthophyll-cycle-dependent quenching are established types of non-photochemical quenching.

### The saturating light pulse method

The saturating light pulse method is an experimental approach that has been devised to determine the amount of photochemical quenching. This approach uses saturating light pulses that have been shown to completely reduce the PQ pool in plants and green algae. In this condition, no electron acceptors are available for Q_A_, and consequently every Q_A_ will be reduced, thus eliminating all photochemical quenching. For work in plants and green algae a standard nomenclature for different fluorescence levels has been adopted. The maximum fluorescence yield that can be achieved during a bright light pulse is termed F_m_. In contrast, the lowest possible fluorescence yield, F_0_ (both F_m_ and F_0_ are defined in dark, aerobic conditions), is the ground fluorescence of the light harvesting complexes of the system recorded in measuring light, which is weak enough to not cause any reduction of Q_A_. During measuring light, the PQ pool stays largely oxidized.

The saturating light pulse method is also able to assess non-photochemical quenching. For this purpose, a reference condition is chosen where non-photochemical quenching is absent. In plants and algae, this is the case in dark and oxygenated conditions, where light harvesting complexes have been shown to be associated with PSII, and the xanthophyll cycle is not engaged. After exposure to light or other conditions known to modulate chlorophyll fluorescence, the decrease in chlorophyll fluorescence induced by saturating light pulses is interpreted as NPQ.

The saturation pulse method has also been applied to heterokont algae, such as *Phaeodactylum* [13, 14] or *Nannochloropsis* [15]. However, the suitability of applying this method in *Nannochloropsis* has not been tested in detail.

### Saturation pulse method in *Nannochloropsis*

In this report, we investigate if the saturation pulse method can be applied to the heterokont alga *Nannochloropsis*. This alga has emerged as a model organism due to its ability to produce triacylglycerols and ω3-fatty acids [16, 17], as well as pigments and antioxidants [18].

The photosynthetic machinery of *Nannochloropsis* is similar to other heterokont algae. However, some *Nannochloropsis*-specific features in the pigmentation and photosystem ratio have been recognized. Unlike many other heterokont algae, which utilize both chlorophyll *a* and *c* in their light harvesting complexes, *Nannochloropsis* only possesses chlorophyll *a* [19]. Also, contrary to green algae and plants, where the ratio between PSI and PSII has been shown to lie between 0.53–0.67 [20], the PSI to PSII ratio in *Nannochloropsis* is about 1.7 [21]. This high ratio of PSI to PSII may make it difficult to fully reduce the PQ pool by bright light pulses used in the saturating light pulse method.

## Materials and methods

### Preparation of cell cultures

*Nannochloropsis oceanica* CCMP1779 obtained from the National Center for Marine Algae and Microbiota (NCMA) was utilized in the study. Pre-cultures were incubated at 18°C and a light intensity of 200 μmol photons m^-2^s^-1^. In all liquid cultures, f/2 medium was utilized [22].

For experiments, cultures with cells in the exponential growth phase were harvested and concentrated to a chlorophyll concentration of 40 μg Chl *a*/ml.

The samples were dark acclimated for three hours prior to starting PAM measurements.

*Chlamydomonas reinhardtii* was included in certain measurements. The strain CC-4532 mt- obtained from the *Chlamydomonas* Resource Center at the University of Minnesota was utilized. TrisAcPO_4_ (TAP) medium was utilized for the liquid *Chlamydomonas* cultures. The pre-cultures of *C*. *reinhardtii* were incubated in the same conditions as the pre-cultures of *Nannochloropsis*, and a chlorophyll *a* concentration of 40 μg/ml was used for experiments. *Chlamydomonas* samples were dark-acclimated one hour prior to measurements.

### PAM fluorometry

For the PAM measurements, a MultiColor PAM fluorometer (Walz, Germany) coupled to an Oxygraph oxygen measurement device (Hansatech, United Kingdom) was used. A volume of 2 ml of cell sample was used for every measurement. During measurements, 0.8 second long bright light pulses of 1600 μmol photons m^-2^s^-1^ of blue light (440 nm) were applied every minute. In the result part, data from every fourth measurement is shown. Our experimental setup for measuring oxygen and chlorophyll fluorescence simultaneously, allowed usage of light intensities as high as 1600 μmol photons m^-2^s^-1^. The MultiColor PAM instrument used also enabled us to choose between saturating light pulses of white and blue light. To assure that the bright light pulses used were saturating (a saturating light pulse is usually defined as a white light pulse having a light intensity above 2000 μmol photons m^-2^s^-1^), we chose to use light pulses of blue light, having a wavelength that matches the 440 nm absorption peak of chlorophyll *a*. Plants and alga absorb only about half the photons in white light, due to the absorption properties of their pigments. The effective light intensity of blue light is therefore about twice as high as white light. The intensity of blue light used in these experiments is consistent with the intensity required to achieve saturating light pulses.

Experiments investigating the effects of anaerobic conditions and high light with and without the use of a *b*_6_*f* complex inhibitor were performed with the setup combining PAM measurements and oxygen measurements. Since this experiment setup restricted the intensity of the bright light pulses to 1600 μmol photons m^-2^s^-1^, an additional experiment was conducted investigating ten bright light pulse intensities ranging from 715 to 5910 μmol photons m^-2^s^-1^. This experiment was performed in aerobic conditions, allowing a different setup where the illumination source could be moved closer to the sample, yielding a higher maximum light intensity of the bright light pulses.

The inhibitor 2,5-dibromo-3-methyl-6-isopropylbenzoquinone (DBMIB) (stock solution: 20 mM) was added to a final concentration of 20 μM.

The cells were left in complete darkness for 12 minutes before DBMIB was added. After 12 more minutes, actinic white light (960 μmol photons/m^2^s) was turned on for 32 minutes. The same actinic light intensity was used for illuminating untreated cells. To obtain anaerobic conditions, cells were placed into the sealed chamber of the oxygen electrode, with respiration in the dark leading to oxygen depletion.

### Processing of PAM data

The raw data was extracted from the MultiColor PAM and the Oxygraph software packages, and MATLAB was utilized to normalize and plot the data. To facilitate comparison of fluorescence signals between the different experiments, the raw data was normalized (the F_0_ value prior to the first bright light pulse was set to zero, while the averaged F_m_ values of the first group of pulses in each experiment, were set to 100).

The variable fluorescence kinetics after bright light pulses were analysed to gain insights into the reduction state of the PQ pool. For this the rate constant (λ) of the chlorophyll fluorescence signal after a bright light pulse (A(t)) was determined by fitting the data to an exponential decay function with baseline offset (y) using MATLAB.

$$A\left(t\right)=A_{0}\cdot e^{-\lambda t}+y$$ (1)

## Results and discussion

In this study, we investigated if the saturation pulse methodology can be applied to the heterokont model alga *Nannochloropsis*. In particular, we were interested if the bright pulses of light intended to induce maximum fluorescence yield (F_m_) has the same effect in *Nannochloropsis* as in green algae and plants.

### Saturating light pulse analysis under different conditions

**Fig 1:**
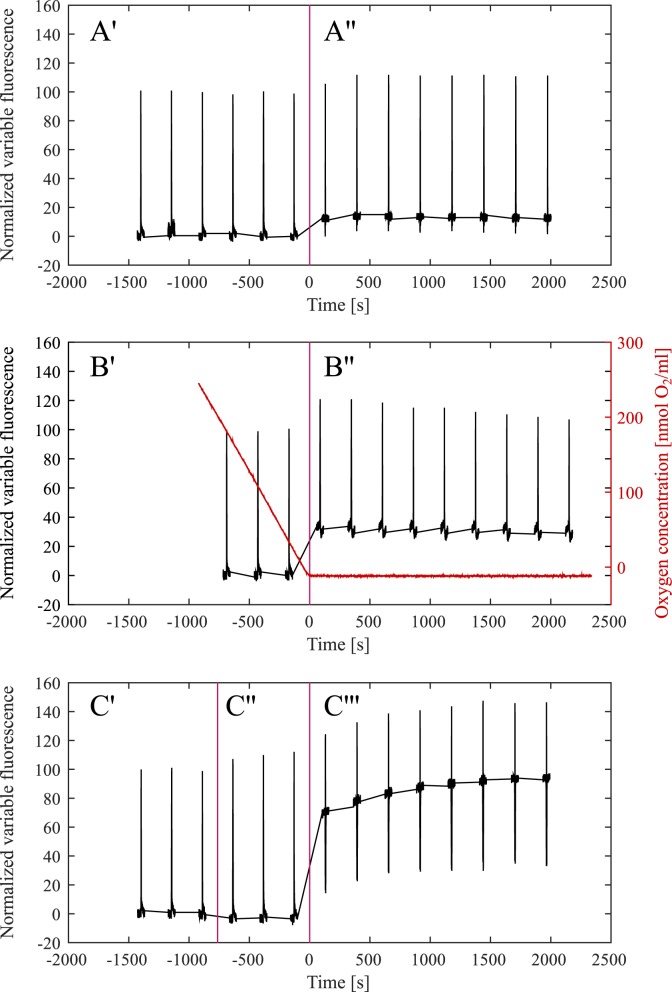
Variable chlorophyll fluorescence assessed by the saturation pulse method in *Nannochloropsis* cells in A) Actinic light (960 μmol photons m^-2^s^-1^), B) Anaerobic conditions, and C) Actinic light after treatment with the cytochrome *b*_6_*f* complex inhibitor DBMIB. Saturating light pulses were applied every 4 minutes. Panel A, split by a vertical line shows the data recorded in darkness (A’), and the data recorded in the presence of high actinic light (A”). The zero time point is defined as the time point when the actinic light was turned on. Panel B, also split by a vertical line, shows the data recorded when oxygen was still available to the cells (B’), and the data recorded after the cells entered anaerobiosis (B”). The red graph shows the oxygen concentration throughout the experiment, and the zero time point is defined as the moment when the oxygen was depleted. Panel C, split by two vertical lines, shows the fluorescence data recorded in darkness before the addition of DBMIB (C’), in darkness after the addition of DBMIB (C”) and after the application of high actinic light (C”‘). The zero time point is defined as the moment when the actinic light was turned on.

To investigate the effect of bright light pulses in *Nannochloropsis* we selected parameters routinely used for saturating light pulse analysis in plants and green algae. We chose light pulses with a length of 0.8 s and an intensity of 1600 μmol of 440 nm (blue) photons m^-2^s^-1^. To assess if these pulses fully reduce the PQ pool we investigated three conditions known to completely reduce the PQ pool in plants and green algae. These conditions include (A) high light treatment (B) anaerobic conditions and (C) blocking the photosynthetic electron transport chain by utilizing the cytochrome *b*_6_*f* complex inhibitor DBMIB (Fig 1).

**Fig 2:**
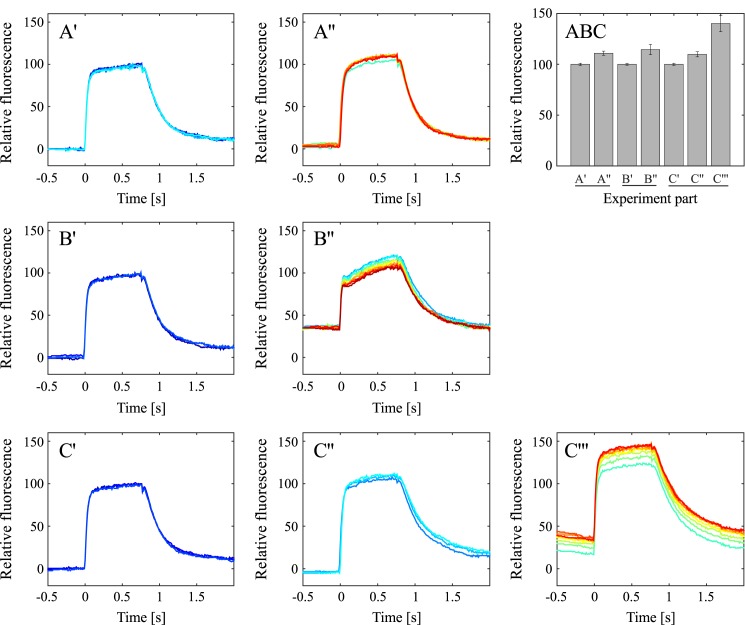
Variable chlorophyll fluorescence emitted by *Nannochloropsis oceanica* cells during bright light pulses. Graph series A shows the high light experiment performed without DBMIB (A’–fluorescence signal of dark adapted cells in darkness, A”–fluorescence signal in high light). Graph series B shows the anaerobic experiment (B’–fluorescence signal of aerobic cells, B”–fluorescence signal of anaerobic cells). Graph series C shows the results from the high light experiment performed in the presence of DBMIB (C’–fluorescence signal of dark-adapted cells in darkness without inhibitor, C”–fluorescence signal of cells in darkness after the addition of DBMIB, C”‘–fluorescence signal of cells in high light, and in the presence of DBMIB). In both experiment A, B and C, the colour of the fluorescence curve indicates when in the experiment the particular curve was recorded. The colours of the fluorescence curves proceed from dark blue (the first recorded curves) via light blue, green, yellow and orange to red (the last recorded curves). The bar diagram (ABC) displayed in the upper right panel summarizes the F_m_ values in the different phases of the three experiments.

Following the standard methodology, light pulses in dark-adapted samples were used to assess the maximum fluorescence yield in the absence of non-photochemical quenching (Fig 1A’,B’ and 1C’). The fluorescence yield of these saturating light pulses is marginally lower than the fluorescence yield observed in high light conditions (Fig 1A”). In anaerobic conditions (Fig 1B”), the maximum fluorescence initially increases, and then decreases again over time. The biggest increase in pulse-induced fluorescence is seen in the presence of DBMIB in high light (Fig 1C”‘). Normalized fluorescence yields from the experiments summarized in Fig 1 are displayed in Fig 2.

The maximum fluorescence levels in the *Nannochloropsis* experiments displayed in Fig 1 follow a different pattern compared to well-documented observations from plants and green algae. In these organisms, bright light pulses induce the maximum fluorescence yield in dark aerobic conditions, while reduction of the PQ pool (through light or anaerobic conditions) leads to NPQ and a reduced light pulse-induced maximum fluorescence. In *Nannochloropsis*, light pulse induced fluorescence yields that are higher than the ones observed in dark-adapted, aerobic conditions can be achieved through actinic light and anaerobic conditions.

The low fluorescence yield in dark aerobic conditions may indicate the presence of a fluorescence quenching mechanism, which is diminished in high light conditions, anaerobic conditions and especially in the presence of DBMIB. This fluorescence quenching may occur within the light harvesting complexes, or may also be caused by an incomplete reduction of the primary fluorescence modulator Q_A_ in dark aerobic conditions, due to an incomplete reduction of the PQ pool. In order to gain further insight into the reduction state of the plastoquinone pool of *Nannochloropsis* in different experimental conditions, the kinetics of change in variable chlorophyll fluorescence after bright light pulses were investigated.

The fluorescence level after 10 ms of bright illumination (commonly referred to as the I level in the OIDP nomenclature [23]) is thought to reflect the accumulation of electrons on Q_A_. This initial level is quite similar in the dark conditions of all three experiments (Fig 2A', 2B' and 2C', and also in high light, anaerobic conditions and in the presence of DBMIB in darkness. Only in DBMIB-treated cells in high light is this initial fluorescence level visibly elevated (Fig 2C‴).

There are two models to explain the fast-rise (O to the I level) and the subsequent slower phases (IDP) of chlorophyll fluorescence induction. The first model assumes the existence of different populations of PSII that differ in their ability to transfer electrons from Q_A_ to Q_B_ [24]. The basis for the difference in Q_A_ to Q_B_ transfer could be an intrinsic property of PSIIs or be based on differences in accessibility to oxidized PQ. The second model used to explain the two phases of the fluorescence induction assumes that the reduction of Q_A_ is the cause of the initial fast fluorescence increase (O to I), whereas the reduction of the plastoquinone pool leads to the subsequent slow increase in fluorescence (I to P). The second model assumes that oxidized plastoquinones quenches fluorescence, most likely via transient electron transfer between oxidized plastoquinones and chlorophyll *a* [10]. Light-induced reduction of the plastoquinone pool requires movement of plastoquinones through the thylakoid membrane and docking of oxidized plastoquinones to PSII prior to electron transfer from Q_A_ [25]. The second phase being slow and showing temperature dependence is in line with the second model [26].

Common to both models is that they both predict that a fully reduced PQ pool will abolish fluorescence quenching. The saturation pulse method uses bright light pulses to reduce the PQ pool and thereby determine the maximum fluorescence yield. However, the fluorescence induction kinetics observed during bright light pulses in *Nannochloropsis* in darkness and in high light (Fig 2) do not show a typical OIDP transient. The lack of fluorescence increase beyond the first inflection point of the fluorescence induction curve (the I level in plants) can be interpreted as the PQ pool already being reduced at this inflection point, or that the PQ pool remains oxidized during the bright light pulse. Anaerobic conditions, which are known to reduce the PQ pool in a variety of organisms, reveal an OIDP transient that is similar to the one observed in plants, thus indicating that the PQ pool is oxidized in aerobic conditions, and can be reduced by bright light pulses in anaerobic conditions. Chlorophyll fluorescence kinetics in dark-adapted cells in the presence of DBMIB do not possess a typical OIDP transient. There is also no OIDP transient in DBMIB-treated and in highlight-treated cells. Surprisingly, the lack of the OIDP transient in DBMIB-treated cells could indicate that the PQ pool is not reduced by saturating light pulses, even if electron transport through the cytochrome *b*_6_*f* complex is blocked.

### Fluorescence decay kinetics as indicator of PQ reduction

To gain insights into the reduction state of the PQ pool after bright light pulses, the decrease in chlorophyll fluorescence immediately following the pulses was analysed. After bright light pulses, the reduced Q_A_^-^ is re-oxidized, leading to a gradual decrease in chlorophyll fluorescence over time. In plants and green algae the oxidation of Q_A_^-^ is due to different processes that can be kinetically grouped within three time domains.

Post-illumination oxidation of Q_**A**_^-^ in the fastest time domain is dependent of the availability of oxidized Q_B_ that is present immediately after a bright light pulse. This Q_B_-dependent Q_A_^-^ oxidation is based on (a) the oxidation of Q_A_^-^ by Q_B_ or Q_B_^-^ (half time ~300 μs, [27, 28]), (b) the exchange of Q_B_^2-^ with an oxidized PQ (half time of electron transport from Q_A_ to Q_B_ when Q_B_ has to bind first ~1,3–2 ms [27]), and (c) the diffusion of oxidized PQ between the cytochrome *b*_6_*f* complex and PSII (half time ~15 ms [28]). Consequently, the decrease in chlorophyll fluorescence due to these fast Q_A_^-^ oxidation events is mostly completed after 50 ms.

Post-illumination oxidation of Q_**A**_^-^ in the second fastest time domain (half time 200 ms) is due to recombination of Q_A_^-^ with the donor side of PSII. It has been demonstrated that the kinetics of Q_A_^-^ oxidation by donor-side electron acceptors is dependent on the redox state of the PQ pool [29] and independent of oxygen concentration [30].

Post-illumination oxidation of Q_**A**_^-^ the third fastest time domain (half time of several seconds) is due to the oxidation of the PQ pool by oxygen-dependent consumption of electrons [30]. The responsible oxidases may be located in the chloroplast (e.g. the plastid terminal oxidase, abbreviated PTOX), or coupled by electron shuttles to the cytoplasmic and mitochondrial electron transport chain [31].

**Fig 3:**
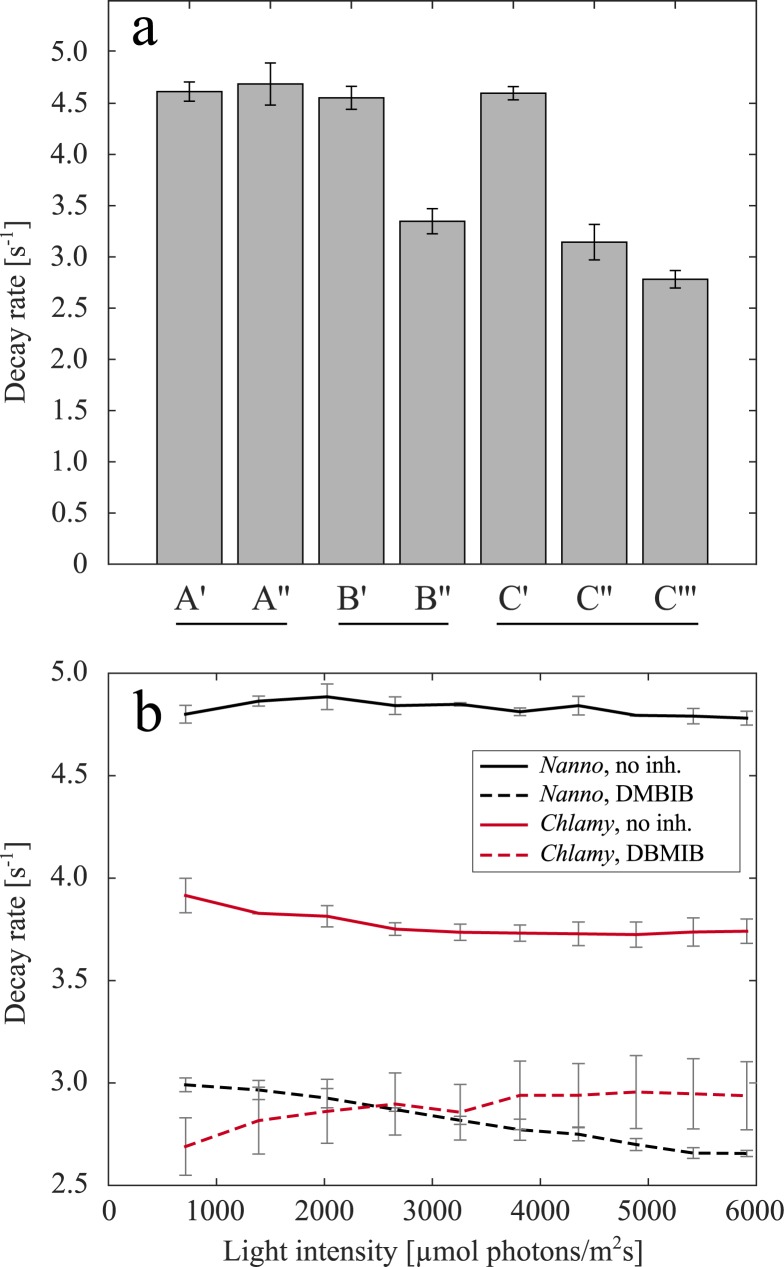
**(a)** Rate constants of the chlorophyll fluorescence decay after a bright light pulse for all phases of the three experiments performed. Averages of the rate constants after bright light pulses displayed in the different groups in Fig 2 are shown. A shows the average of decay rates in the high light experiment performed without DBMIB (A’–fluorescence signal of dark adapted cells in darkness, A”–fluorescence signal in high light). B shows the anaerobic experiment (B’–fluorescence signal of aerobic cells, B”–fluorescence signal of anaerobic cells). Graph series C shows the results from the high light experiment performed in the presence of DBMIB (C’–fluorescence signal of dark-adapted cells in darkness without inhibitor, C”–fluorescence signal of cells in darkness after the addition of DBMIB, C”‘–fluorescence signal of cells in high light, and in the presence of DBMIB). **(b)** Decay rates of chlorophyll fluorescence kinetics after bright light pulses in *Nannochloropsis oceanica* and *Chlamydomonas reinhardtii* without and with the addition of DBMIB under varying intensities of bright light pulses.

We have analysed the chlorophyll fluorescence decrease in the second fastest phase, which–in analogy to higher plants -, we assume to rely on the recombination of Q_A_^-^ with the donor side of PSII, to gain insights into the reduction state of the PQ pool after saturating light pulses. Fig 3A shows rate constants for the fluorescence decrease after bright light pulses in different conditions (fluorescence curves displayed in Fig 2). The fit parameters obtained for fitting Eq 1 to fluorescence transients after a light pulse can be found in S1 and S2 Tables.

Subjecting the cells to anaerobic conditions and treatment with DBMIB (in dark-adapted conditions and high light) leads to a significant decrease in the decay constants compared to dark aerobic conditions (Fig 3A). This distribution of rate constants indicate that the PQ pool has a higher degree of reduction after saturating light pulses in anaerobic conditions and in the presence of DBMIB (dark-adapted and high light) compared to aerobic conditions (dark-adapted and high light). We therefore concluded that bright light pulses with an intensity of 1600 μmol photons m^-2^s^-1^ of blue light (440 nm) leave the PQ pool oxidized.

The apparent inability of bright light pulses to reduce the PQ pool in standard (dark aerobic) conditions was further investigated. For this we varied the light pulse intensity (from 715 to 5910 μmol photons m^-2^s^-1^) and analysed the rate constants of the fluorescence curves in aerobic dark conditions in the presence and absence of DBMIB (Fig 3B). We also determined the fluorescence kinetics subsequent to bright pulses of light in the green alga *Chlamydomonas* under the same conditions.

Increasing the light intensity does not decrease the rate constants in untreated *Nannochloropsis* cells, while a light-dependent decrease in rate constants for *Chlamydomonas* can be observed. The rate constants for untreated *Nannochloropsis* cells are higher than the rate constants for untreated *Chlamydomonas* cells. This indicates that even light pulses with very high light intensity are unable to reduce the PQ pool in *Nannochloropsis*.

Addition of DBMIB reduces the rate constants after bright light pulses in *Nannochloropsis* and *Chlamydomonas* to a similar level. This data indicate that in the presence of DBMIB, the PQ pool of both *Nannochloropsis* and *Chlamydomonas* PQ is reduced after the application of bright light pulses. This would be expected as DBMIB is thought to block electron transport at the cytochrome *b*_6_*f* complex.

## Conclusion and implications

### Effect of bright light pulses

According to our interpretation of the fluorescence data, bright light pulses of 0.8 s are not sufficient to reduce the PQ pool in untreated *Nannochloropsis oceanica* cells.

The most likely reason for this is the unusually high PSI:PSII ratio of about 1.7 [21, S1 Fig] in *Nannochloropsis*. In plants, the PSI:PSII ratio has been found to lie between 0.53 and 0.67 [20]. The excess of PSI in *Nannochloropsis* may therefore be able to maintain a partially oxidized PQ pool, even during bright illumination.

Our finding that the PQ pool in *Nannochloropsis* is not reduced by bright light pulses has implications for interpreting results obtained by the saturating light pulse method, since a fully reduced plastoquinone pool is a prerequisite for the application of saturating light pulse analysis. Therefore, caution should be exercised when the saturating light pulse method is applied to assess photosynthetic performance in *Nannochloropsis*.

### The lack of a traditional plant OIDP transient

Our experiments in anaerobic conditions show a conventional OIDP transient, while in aerobic conditions, no IDP transient can be distinguished. We can think of two likely reasons for the presence of the OIDP transient in anaerobic conditions. (1) One or more oxygen-dependent electron transport pathways may oxidize PQ, but this electron sink is lost in anaerobic conditions. The oxygen-dependent electron sink could consist of a Mehler-type reaction [32] or be mediated by a chloroplast-located oxidase. Analysis of the *Nannochloropsis gaditana* genome indicates the presence of a plastid terminal oxidase (PTOX) homologue. (2) With a limited amount of possible electron acceptors present in anaerobic conditions, *Nannochloropsis* PSI may exhibit variable fluorescence, depending on electron acceptors within PSI becoming reduced. In plants and algae it is usually assumed that PSI does not possess variable fluorescence, and only contributes to the base fluorescence level (F_0_). This may be different in *Nannochloropsis*. Detailed fluorescence emission spectra may be utilized to discern a possible PSI-dependent variable fluorescence in future studies.

### Effect of DBMIB

Rate constants of fluorescence kinetics after bright light pulses indicate that DBMIB blocks electron transport in *Nannochloropsis*. As in plants and algae, the cytochrome *b*_6_*f* complex is the likely target for DBMIB. Interestingly, high light-induced reduction of the PQ pool in DBMIB-treated *Nannochloropsis* cells appears to abolish one or more chlorophyll fluorescence quenching mechanisms within minutes. A plant-derived interpretation of the observed increase in maximum chlorophyll fluorescence is that PSII is gaining more excitation. This interpretation is counter-intuitive for DBMIB-treated *Nannochloropsis*, as the correct physiological response would be to avoid electron generation by PSII when the PQ pool is reduced. Alternatively, the increase in fluorescence in high light and DBMIB-treated *Nannochloropsis* cells may indicate PSII damage. In any case, the high light-induced changes in DBMIB-treated cells serves as a reminder that plant-based interpretations may not be suitable for interpreting chlorophyll fluorescence in *Nannochloropsis*.

### Photoprotection in *Nannochloropsis*

The inability of high light to induce a reduced PQ pool in *Nannochloropsis* is also of relevance for investigating photoprotection mechanisms in this alga. The most prominent photoprotection mechanisms observed in plants and green algae are the xanthophyll cycle and state transitions. In plants and green algae, state transitions are initiated by a reduced PQ pool [4], while the xanthophyll cycle is initiated by a low pH in the thylakoid lumen [33]. However, in *Nannochloropsis* it is unlikely that high light induces state transitions, as the PQ pool remains partially oxidized. This might be one reason why state transitions so far have not been detected in *Nannochloropsis*. Furthermore, electron transport through the PQ pool and concomitant proton translocation occurs even at high light intensities in *Nannochloropsis*. Therefore the pH-dependent xanthophyll cycle is likely more easily induced in *Nannochloropsis* than in green algae and plants, where electron transport stalls during high light illumination. When investigating mechanisms involved in photoprotection, the most common method for reducing plastoquinone is to subject the cells to high light, usually not in the presence of DBMIB [15, 34]. Thus, an oxidized PQ pool under high light intensities is in line with the observed predominance of the xanthophyll cycle as a photoprotection mechanism in *Nannochloropsis*.

## Supporting information

S1 Fig 77K spectrum of *Nannochloropsis*, indicating a high ratio of PSI:PSII. 77K spectroscopy spectrum of *Nannochloropsis* cells normalized to a chlorophyll concentration of 2.5 μmol chlorophyll ml^-1^. The spectrum is normalized to 1.
(PNG)

S1 Table Fluorescence decay parameters. Parameters resulting from the fitting of Eq 1 to the decay part (0.8–3 s) of the fluorescence curves shown in Fig 2. The amplitudes (A_0_), decay rates (λ), constants (y) and R^2^ values are shown as averages for the parts of the different experiments. Group A’ and A” correspond to the groups of fluorescence curves recorded in darkness (A’) and high light (A”) in the high light experiment performed without DBMIB. Group B’ and B” correspond to the groups of fluorescence curves recorded in aerobic conditions (B’) and anaerobic conditions (B”) in the anaerobic experiment performed in darkness. Group C’, C” and C”‘ correspond to the groups of fluorescence curves recorded in darkness without inhibitor (C’), in darkness after the addition of DBMIB (C”), and in high light (C”‘) in the high light experiment performed with DBMIB.
(CSV)

S2 Table Fluorescence decay parameters. Standard deviations within the groups of variables that were averaged to obtain the parameters shown in table S1 Table.
(CSV)

## Abbreviations

DBMIB: 2,5-dibromo-3-methyl-6-isopropylbenzoquinone
LHCII: Light Harvesting Complex II
NPQ: Non-photochemical Quenching
PAM: Pulse Amplitude Modulation
PQ: Plastoquinone
PSI: Photosystem I
PSII: Photosystem II
PTOX: Plastid terminal oxidase

## References

[1] Beer S, Björk M. Measuring rates of photosynthesis of two tropical seagrasses by pulse amplitude modulated (PAM) fluorometry. Aquat Bot. 2000;66:69–76.

[2] White S, Anandraj A, Bux F. PAM fluorometry as a tool to assess microalgal nutrient stress and monitor cellular neutral lipids. Bioresource Technol. 2011;102:1675–1682. 10.1016/j.biortech.2010.09.097 20965719

[3] Baker NR. Chlorophyll fluorescence: A probe of photosynthesis in vivo. Annu Rev Plant Biol. 2008;59:89–113. doi: 10.1146/annurev.arplant.59.032607.092759 18444897 10.1146/annurev.arplant.59.032607.092759

[4] Haldrup A, Jensen PE, Lunde C, Scheller HV. Balance of power: a view of the mechanism of photosynthetic state transitions. Trends Plant Sci. 2001;6:301–305. 11435168 10.1016/s1360-1385(01)01953-7

[5] Finazzi G, Rappaport F, Furia A, Fleischmann M, Rochaix J-D, Zito F, et al Involvement of state transitions in the switch between linear and cyclic electron flow in *Chlamydomonas reinhardtii*. EMBO Rep. 2002;3:280–285. doi: 10.1093/embo-reports/kvf047 11850400 10.1093/embo-reports/kvf047 PMC1084013

[6] Kargul J, Turkina MV, Nield J, Benson S, Vener AV, Barber J. Light-harvesting complex II protein CP29 binds to photosystem I of *Chlamydomonas reinhardtii* under state 2 conditions. FEBS J. 2005;272:4797–4806. doi: 10.1111/j.1742-4658.2005.04894.x 16156798 10.1111/j.1742-4658.2005.04894.x

[7] Ünlü C, Drop B, Croce R, van Amerongen H. State transitions in *Chlamydomonas reinhardtii* strongly modulate the functional size of photosystem II but not of photosystem I. P Natl Acad Sci USA. 2014;111:3460–3465. 10.1073/pnas.1319164111 PMC3948275 24550508

[8] Catalanotti C, Yang W, Posewitz MC, Grossman AR. Fermentation metabolism and its evolution in algae. Front Plant Sci. 2013;4:1–17. 23734158 10.3389/fpls.2013.00150 PMC3660698

[9] Green BR, Anderson JM, Parson WW. Photosynthetic membranes and their light-harvesting antennas In: Green BR and Parson WW (ed) Light-harvesting antennas in photosynthesis, 1st edn. Springer Publishers, The Netherlands; 2003 pp 1–28

[10] Haldimann P, Tsimilli-Michael M. Non-photochemical quenching of chlorophyll *a* fluorescence by oxidised plastoquinone: new evidences based on modulation of the redox state of the endogenous plastoquinone pool in broken spinach chloroplasts. Biochim Biophys Acta. 2005;17:239–249. 10.1016/j.bbabio.2004.11.005 15694352

[11] Vernotte C, Etienne AL, Briantais J-M. Quenching of the System II chlorophyll fluorescence by the plastoquinone pool. Biochim Biophys Acta. 1979;545:519–527. 427143 10.1016/0005-2728(79)90160-9

[12] Hohmann-Marriott MF, Takizawa K, Eaton-Rye JJ, Mets L, Minagawa J. The redox state of the plastoquinone pool directly modulates minimum chlorophyll fluorescence yield in *Chlamydomonas reinhardtii*. FEBS Lett. 2010;584:1021–1026. doi: 10.1016/j.febslet.2010.01.052 20122933 10.1016/j.febslet.2010.01.052

[13] Ting CS, Owens TG. Photochemical and non-photochemical fluorescence quenching processes in the diatom *Phaeodactylum tricornutum*. Plant Physiol. 1993;101:1323–1330. 12231788 10.1104/pp.101.4.1323 PMC160656

[14] Liu X, Duan S, Li A, Xu N, Cai Z, Hu Z. Effects of organic carbon sources on growth, photosynthesis, and respiration of *Phaeodactylum tricornutum*. J Appl Phycol. 2009;21:239–246.

[15] Cao S, Zhang X, Xu D, Fan X, Mou S, Wang Y, Ye N, Wang W. A transthylakoid proton gradient and inhibitors induce a non-photochemical fluorescence quenching in unicellular algae *Nannochloropsis* sp. FEBS Lett. 2013;587:1310–1315. doi: 10.1016/j.febslet.2012.12.031 23474242 10.1016/j.febslet.2012.12.031

[16] Lui B, Vieler A, Li C, Jones DA, Benning C. Triacylglycerol profiling of microalgae *Chlamydomonas reinhardtii* and *Nannochloropsis oceanica*. Bioresource Technol. 2013;146:210–316. 10.1016/j.biortech.2013.07.088 23948268

[17] Mitra M, Patidar SK, Mishra S. Integrated process of two stage cultivation of *Nannochloropsis* sp. for nutraceutically valuable eicosapentaenoic acid along with biodiesel. Bioresource Technol. 2015;193:363–369. 10.1016/j.biortech.2015.06.033 26143004

[18] Lubián LM, Montero O, Moreno-Garrido I, Huertas EI, Sobrino C, González-del Valle M, Parés G. *Nannochloropsis* (*Eustigmatophyceae*) as source of commercially valuable pigments. J Appl Phycol. 2000;12:249–255.

[19] Basso S, Simionato D, Gerotto C, Segalla A, Giacometti GM, Morosinotto T. Characterization of the photosynthetic apparatus of the Eustigmatophycean *Nannochloropsis* gaditana: Evidence of convergent evolution in the supramolecular organization of photosystem I. Biochim Biophys Acta. 2014;1837:306–314. doi: 10.1016/j.bbabio.2013.11.019 24321505 10.1016/j.bbabio.2013.11.019

[20] Fan D-Y, Hope AB, Smith PJ, Jia H, Pace RJ, Anderson JM, Chow WS. The stoichiometry of the two photosystems in higher plants revisited. Biochim Biophys Acta. 2007;1767:1064–1072. doi: 10.1016/j.bbabio.2007.06.001 17618597 10.1016/j.bbabio.2007.06.001

[21] Carbonera D, Agostini A, Di Valentin M, Gerotto C, Basso S, Giacometti GM, Morosinotto T. Photoprotective sites in the violaxanthin-chlorophyll *a* binding protein (VCP) from *Nannochloropsis gaditana*. Biochim Biophys Acta. 2014;1837:1235–1246. doi: 10.1016/j.bbabio.2014.03.014 24704151 10.1016/j.bbabio.2014.03.014

[22] Guillard RRL, Ryther JH. Studies of marine planktonic diatoms. I. *Cyclotella nana* Hustedt and *Detonula confervacea* (Cleve) Gran. Can J Microbiol. 1962;8:229–239. 13902807 10.1139/m62-029

[23] Govindjee. Sixty-three years since Kautsky: Chlorophyll *a* fluorescence. Aust J Plant Physiol. 1995;22:131–160

[24] Cao J, Govindjee. Chlorophyll *a* fluorescence transient as an indicator of active and inactive photosystem II in thylakoid membranes. Biochim Biophys Acta. 1990;1015:180–188. 2404518 10.1016/0005-2728(90)90018-y

[25] Stirbet A, Govindjee. Chlorophyll *a* fluorescence induction: a personal perspective of the thermal phase, the J-I-P rise. Photosynth Res. 2012;113:15–61. doi: 10.1007/s11120-012-9754-5 22810945 10.1007/s11120-012-9754-5

[26] Neubauer C, Schreiber U. The polyphasic rise of chlorophyll fluorescence upon onset of strong continuous illumination: I. Saturation characteristics and partial control by the photosystem II acceptor side. Z Naturforsch C. 1987;42c:1246–1254.

[27] De Wijn R, Van Gorkom HJ. Kinetics of electron transfer from Q_A_ to Q_B_ in photosystem II. Biochemistry-US. 2001;40:11912–11922. 10.1021/bi010852r 11570892

[28] Govindjee, Kern JF, Messinger J, Whitmarsh J. Photosystem II In: Encyclopedia of Life Sciences (ELS). John Wiley & Sons, Ltd, Chichester; 2010

[29] Diner BA. Dependence of the deactivation reactions of photosystem II on the redox state of plastoquinone pool A varied under anaerobic conditions; Equilibria on the acceptor side of photosystem II. Biochim Biophys Acta. 1977;460:247–258. 870036 10.1016/0005-2728(77)90211-0

[30] Laisk A, Eichelmann H, Oja V. Oxidation of plastohydroquinone by photosystem II and by dioxygen in leaves. Biochim Biophys Acta. 2015;1847:565–575. doi: 10.1016/j.bbabio.2015.03.003 25800682 10.1016/j.bbabio.2015.03.003

[31] Peltier G, Cournac L. Chlororespiration. Annu Rev Plant Biol. 2002;53:523–550. doi: 10.1146/annurev.arplant.53.100301.135242 12227339 10.1146/annurev.arplant.53.100301.135242

[32] Heber U. Irrungen, Wirrungen? The Mehler reaction in relation to cyclic electron transport in C3 plants. Photosynth Res. 2002;73:223–231. doi: 10.1023/A:1020459416987 16245125 10.1023/A:1020459416987

[33] Jahns P, Holzwarth AR. The role of the xanthophyll cycle and of lutein in photoprotection of photosystem II. Biochim Biophys Acta. 2012;1817:182–193. doi: 10.1016/j.bbabio.2011.04.012 21565154 10.1016/j.bbabio.2011.04.012

[34] Müller P, Li X-P, Niyogi KK. Non-photochemical quenching. A response to excess light energy. Plant Physiol. 2001;125:1558–1566. 11299337 10.1104/pp.125.4.1558 PMC1539381

