# Supplementary figures and images for: The plastoquinone pool of Nannochloropsis oceanica is not completely reduced during bright light pulses
Source: PLoS One. 2017 Apr 12;12(4):e0175184. doi: 10.1371/journal.pone.0175184 (PMC5389811; doi:10.1371/journal.pone.0175184)

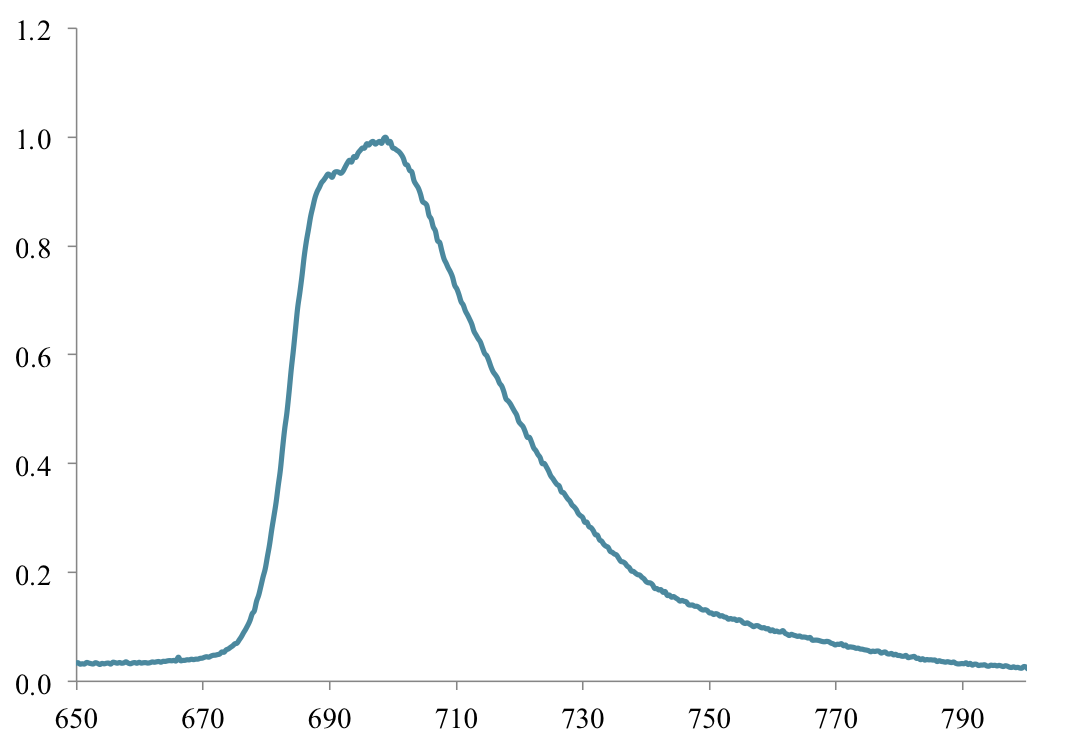

Supplement: S1 Fig — 77K spectroscopy spectrum of Nannochloropsis cells normalized to a chlorophyll concentration of 2.5 μmol chlorophyll ml-1. The spectrum is normalized to 1. (PNG) [file pone.0175184.s001.png]
